# Supplementary material for: Pyrogenic carbon contribution to tropical savanna soil carbon storage
Source: Nat Commun. 2025 Nov 4;16:9730. doi: 10.1038/s41467-025-64699-y (PMC12586565; doi:10.1038/s41467-025-64699-y)
Supplement: Supplementary file 1 — Supplementary Information [file 41467_2025_64699_MOESM1_ESM.pdf]

Supplementary Information *for*

Pyrogenic carbon contribution to tropical savanna soil carbon storage

Yong Zhou [1, 2, *], A. Tyler Karp [3], Abigail Schmidt [1], Corli Coetsee [4, 5]

[1] Department of Wildland Resources and Ecology Center, Utah State University, Logan, Utah, 84321, USA

[2] Department of Ecology, Evolution, and Marine Biology, University of California, Santa Barbara, Santa Barbara, California, 93016, USA

[3] Department of the Geophysical Science, University of Chicago, Chicago, Illinois 60637, USA

[4] School of Natural Resource Management, Nelson Mandela University, George 6529, South Africa

[5] Scientific Services, Kruger National Park, Private Bag X402, Skukuza 1350, South Africa

[*] Correspondence: yongzhou@ucsb.edu

This PDF file includes:

Supplementary Figures 1-10

Supplementary Table 1

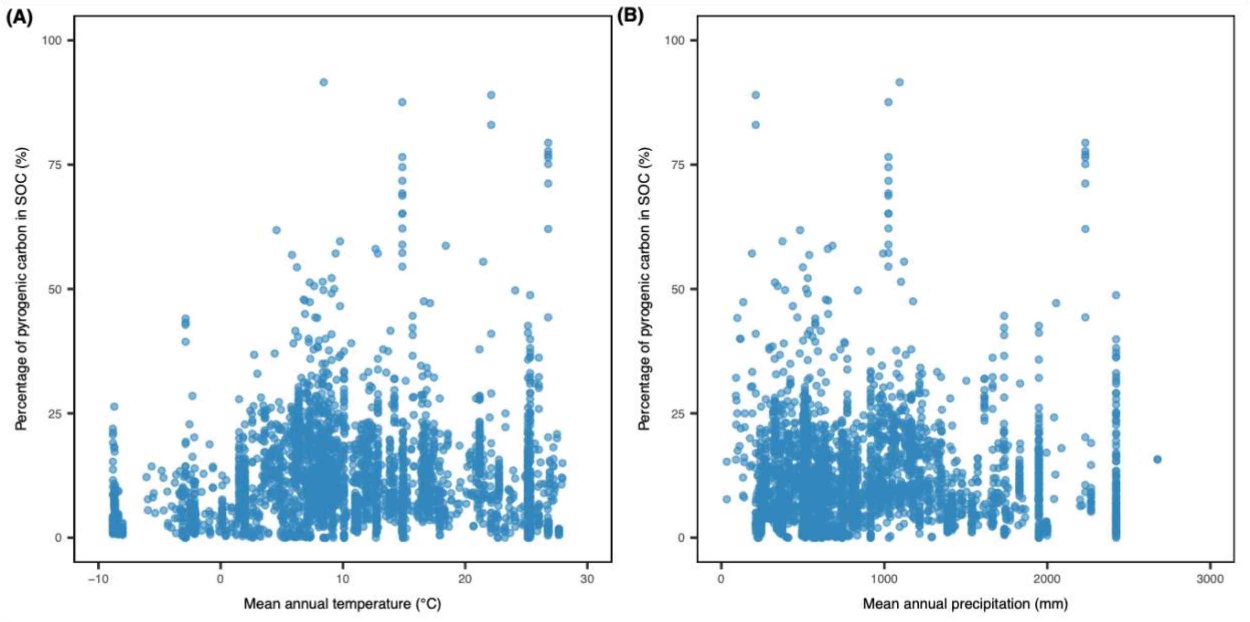

Supplementary Fig. 1: The percentage of pyrogenic carbon in soil organic carbon (SOC, %) was not predicted by mean annual temperature (°C) or mean annual precipitation (mm). (**A**) Scatter plot showing the relationship between the percentage of pyrogenic carbon in SOC (%) and mean annual temperature (°C). (**B**) Scatter plot showing the relationship between the percentage of pyrogenic carbon in SOC (%) and mean annual precipitation (mm). Mean annual temperature and precipitation were extracted from WorldClim 2.0 using site coordinates.

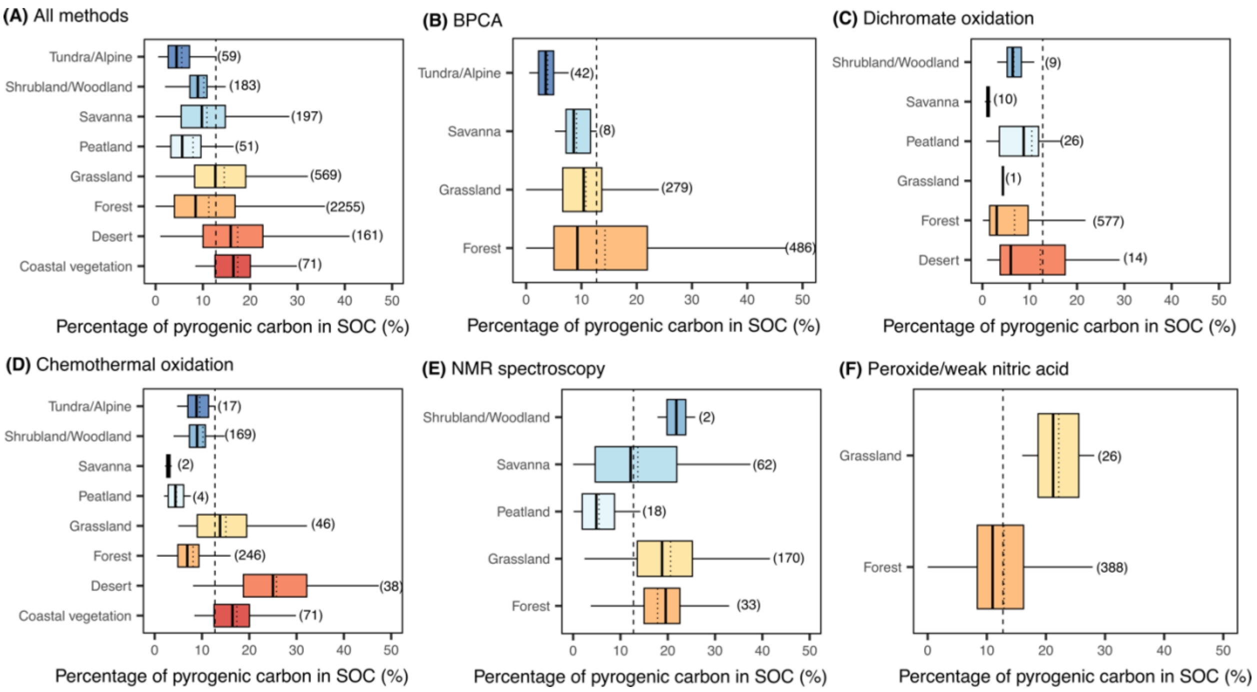

Supplementary Fig. 2: The percentage of pyrogenic carbon in soil organic carbon (SOC) (%) across different ecosystems, measured using multiple methods. Panels show comparisons for all methods combined (**A**), benzene polycarboxylic acid (BPCA) (**B**), dichromate oxidation (**C**), chemothermal oxidation (**D**), nuclear magnetic resonance (NMR) spectroscopy (**E**), and peroxide/weak nitric acid (**F**). Boxes represent the interquartile range (25th to 75th percentiles), with the horizontal line indicating the median. Whiskers extend to the most extreme values within 1.5 times the interquartile range. The dotted line within each box shows the mean value for that ecosystem, while the dashed vertical line represents the mean across all ecosystems. Numbers next to each box indicate the number of measurements for that ecosystem.

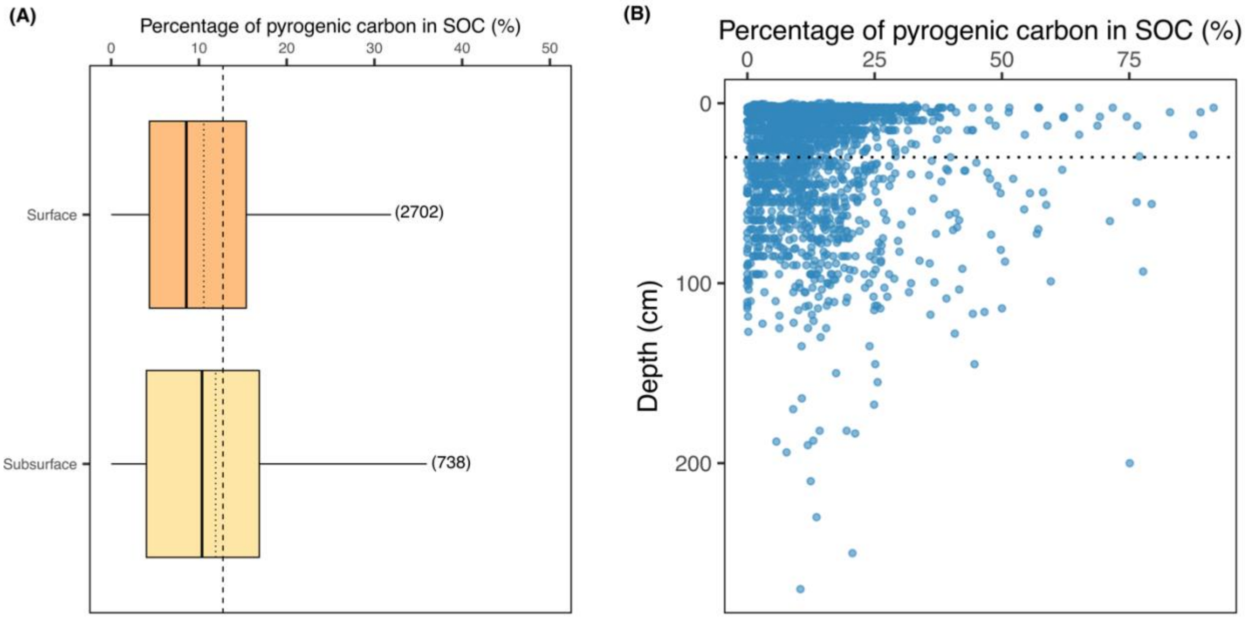

Supplementary Fig. 3: The percentage of pyrogenic carbon in soil organic carbon (SOC) (%) across different soil depths is shown. (**A**) Box plots depict the percentage of pyrogenic carbon in SOC (%) within surface soils (0–30 cm) and subsurface soils (> 30 cm). The boxes represent the interquartile range (25th to 75th percentiles), with the horizontal line indicating the median. Whiskers extend to the most extreme values within 1.5 times the interquartile range. A dotted line within each box shows the mean value for that soil depth, while a dashed vertical line represents the mean across all data. Numbers next to each box indicate the number of measurements for that soil depth. Soil depths that cross the 30 cm threshold (*e.g.*, 10–40 cm) were excluded from this analysis. (**B**) A scatter plot shows the relationship between the percentage of pyrogenic carbon in SOC and soil depth. Soil depths were calculated as the mean of the start and end depths of each depth increment; for example, a depth interval of 0–20 cm is represented as 10 cm.

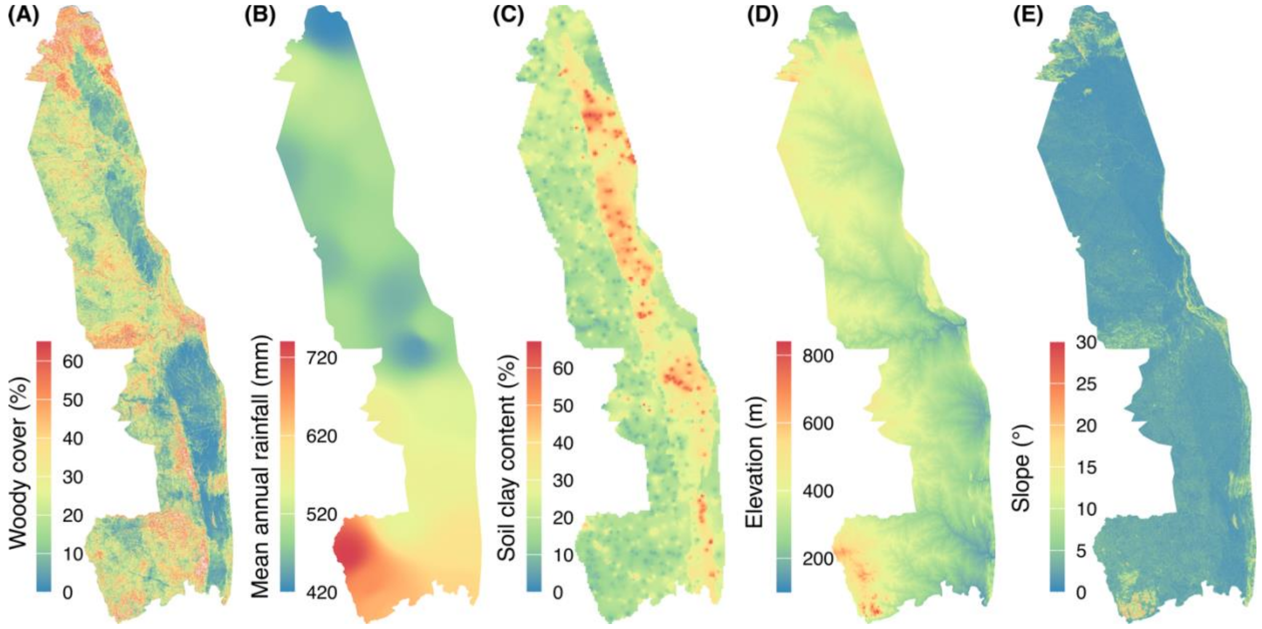

Supplementary Fig. 4: **Explanatory variables used to predict pyrogenic carbon stocks in this study**. Maps showing woody cover (%) (**A**), mean annual rainfall (mm) (**B**), soil clay content (%) (**C**), elevation (m) (**D**), and slope (°) (**E**) across Kruger National Park, South Africa. These maps were used to derive related parameters for 253 Veld Condition Assessment sites in this study. The Kruger National Park shapefile was provided by South African National Parks Scientific Services.

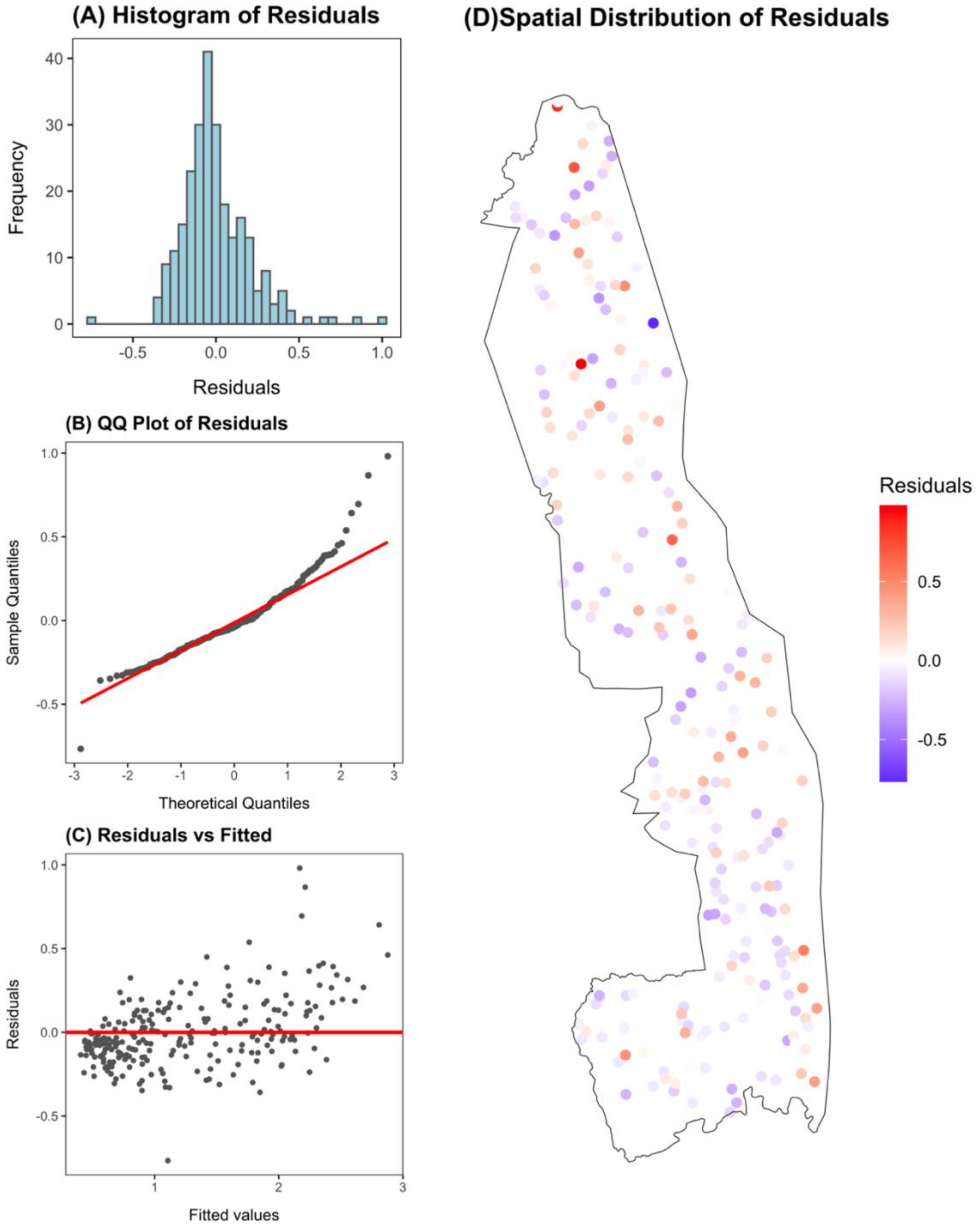

Supplementary Fig. 5: **Model residuals from the optimized random forest model**. (**A**) histogram of residuals, (**B**) QQ plot of residuals, (**C**) residuals vs. fitted values, and (**D**) spatial distribution of residuals. The Kruger National Park shapefile was provided by South African National Parks Scientific Services.

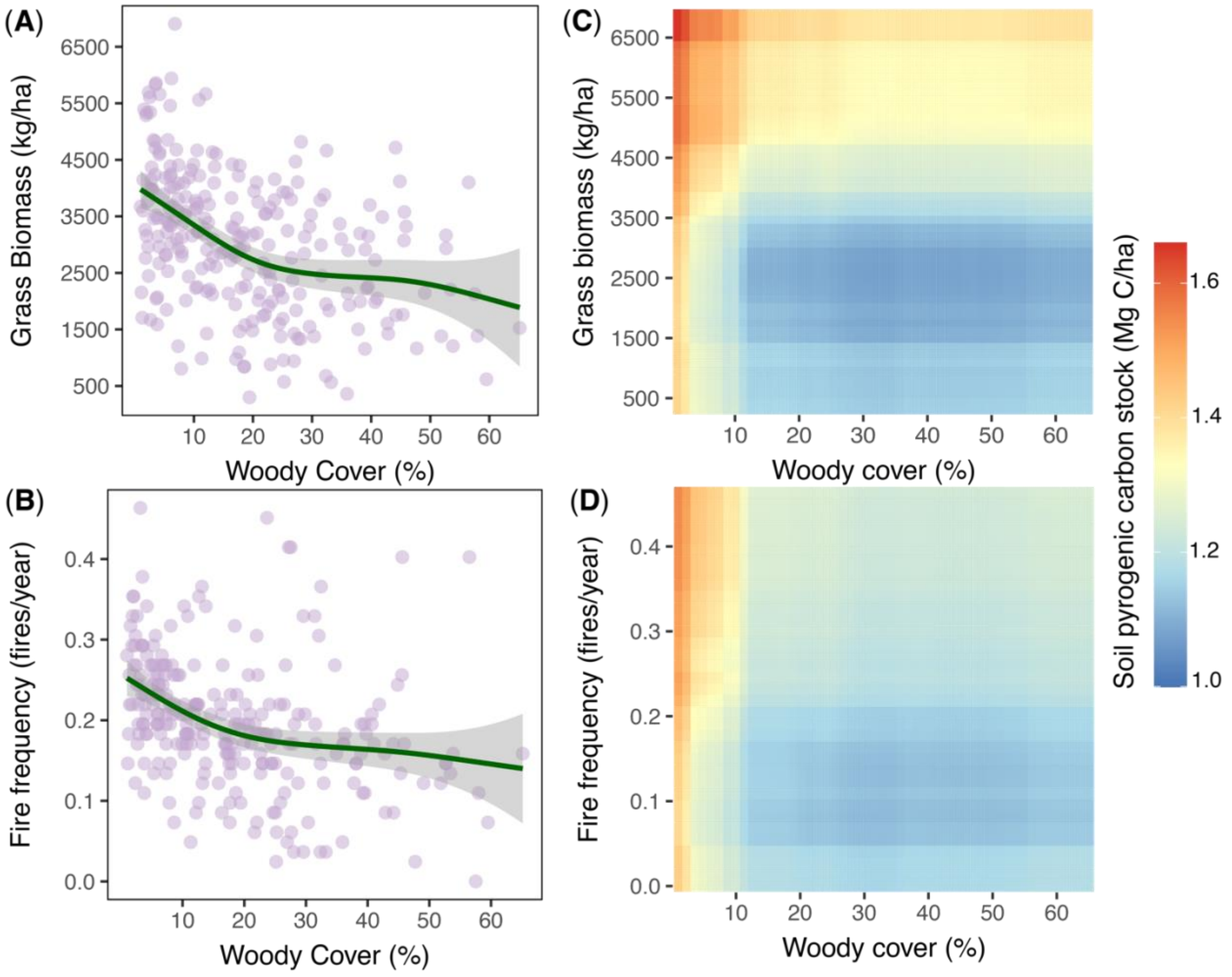

Supplementary Fig. 6: **The relationship between woody cover, grass biomass, fire frequency, and their interactive effects on soil pyrogenic carbon accumulation across Kruger National Park, South Africa**. (**A**) Scatter plot showing the relationship between woody cover (%) and grass biomass (kg/ha). (**B**) Scatter plot showing the relationship between woody cover (%) and fire frequency (fires/year). (**C**) Two-dimensional partial dependence plot illustrating the interactive effects of woody cover and grass biomass on soil pyrogenic carbon stock. (**D**) Two-dimensional partial dependence plot illustrating the interactive effects of woody cover and fire frequency on soil pyrogenic carbon stock. In panels (**A**) and (**B**), the line represents a loess smooth fit, with gray bands indicating the 95% confidence intervals. In panels (**C**) and (**D**), results are derived from the best-fitted random forest model (see Methods for further details).

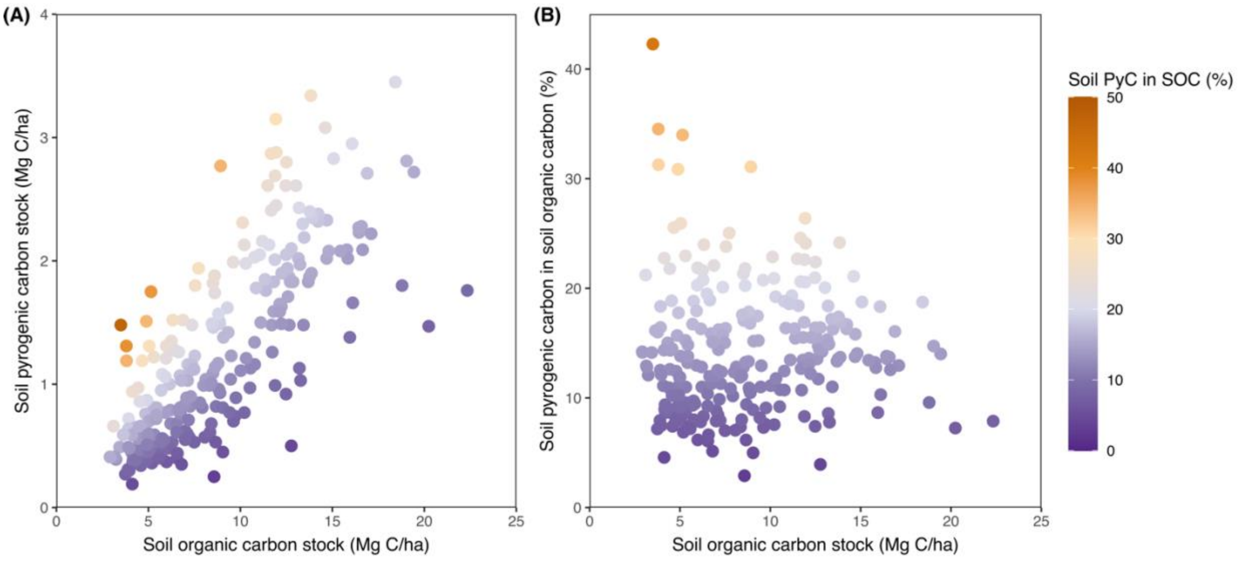

Supplementary Fig. 7: Scatter plots showing the relationships between soil pyrogenic carbon (PyC) stock (Mg C/ha) and soil organic carbon (SOC) stock (Mg C/ha) (A), as well as the percentage of PyC in SOC (%) and SOC stock (Mg C/ha) (B). While soil PyC stock was linearly related to SOC stock ($R^2 = 0.61$, $p < 0.0001$, n = 253), soils with higher SOC stock did not exhibit a higher percentage of PyC in SOC ($R^2 = 0.002$, $p = 0.48$, n = 253).

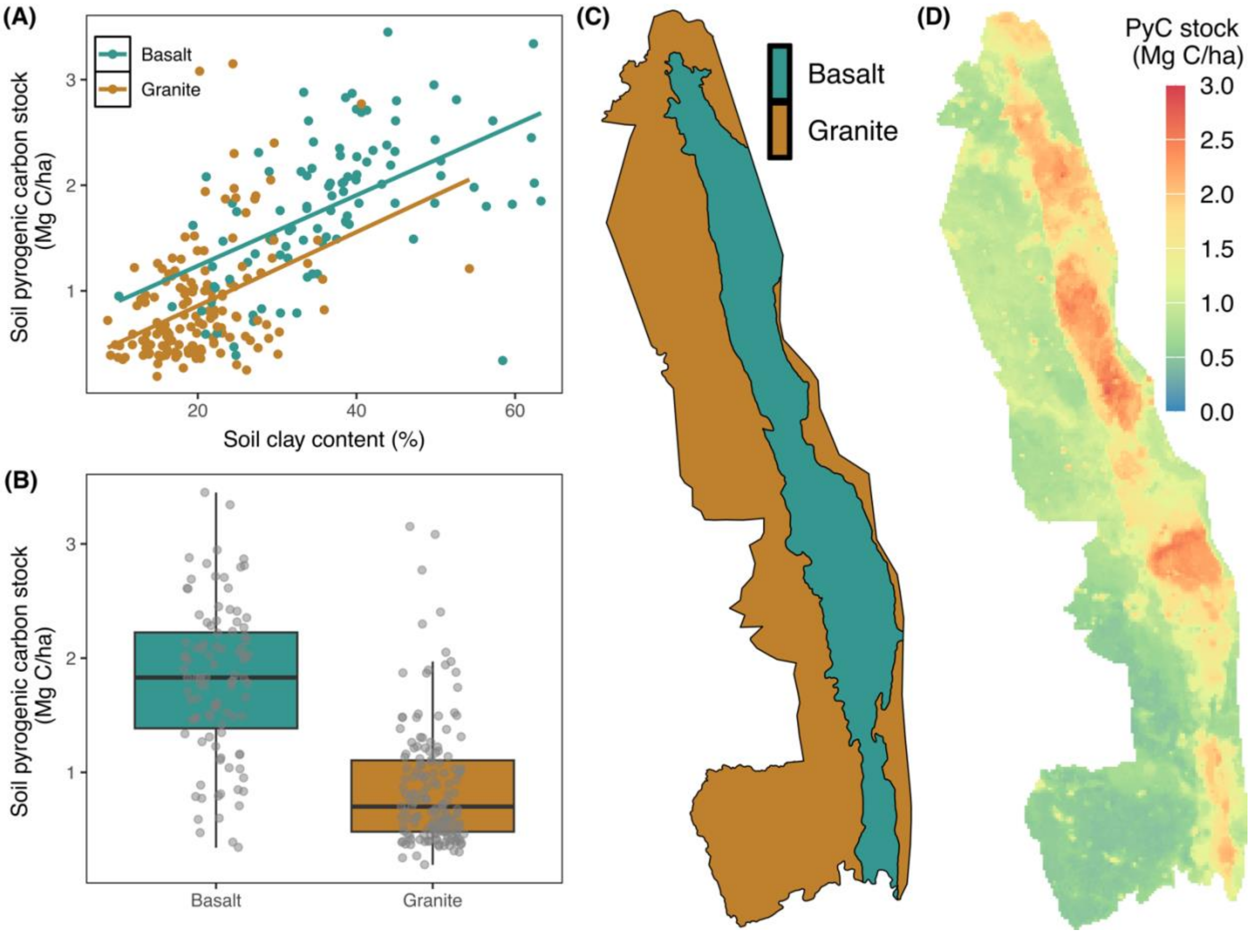

Supplementary Fig. 8: Soil pyrogenic carbon stock and its association with soil parent materials across Kruger National Park. (**A**) Relationship between soil pyrogenic carbon stock (Mg C/ha) and soil clay content based on soil parent materials (basalt vs. granite). (**B**) Boxplot of soil pyrogenic carbon stock (Mg C/ha) for different soil parent materials (basalt vs. granite). (**C**) Distribution of soil parent materials (basalt vs. granite) across Kruger National Park. (**D**) Predicted soil pyrogenic carbon stock across Kruger National Park based on the best-fit random forest model. In panel B, the box represents the interquartile range (25th to 75th percentile), with a horizontal line indicating the median. Whiskers extend to the smallest and largest values within 1.5 times the IQR, while points beyond the whiskers are considered outliers. The Kruger National Park shapefile was provided by South African National Parks Scientific Services.

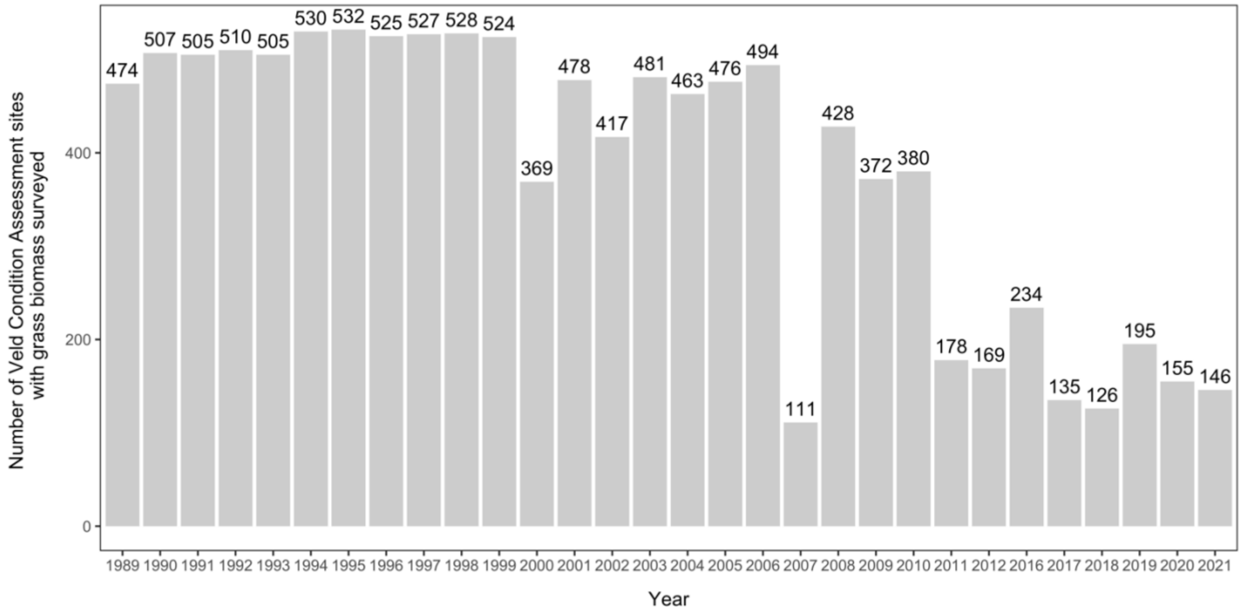

Supplementary Fig. 9: **Number of Veld Condition Assessment sites with grass biomass surveyed from 1989 to 2012 and from 2016 to 2021 in Kruger National Park, South Africa**. These data were used to calculate the long-term grass biomass for 253 Veld Condition Assessment sites in this study.

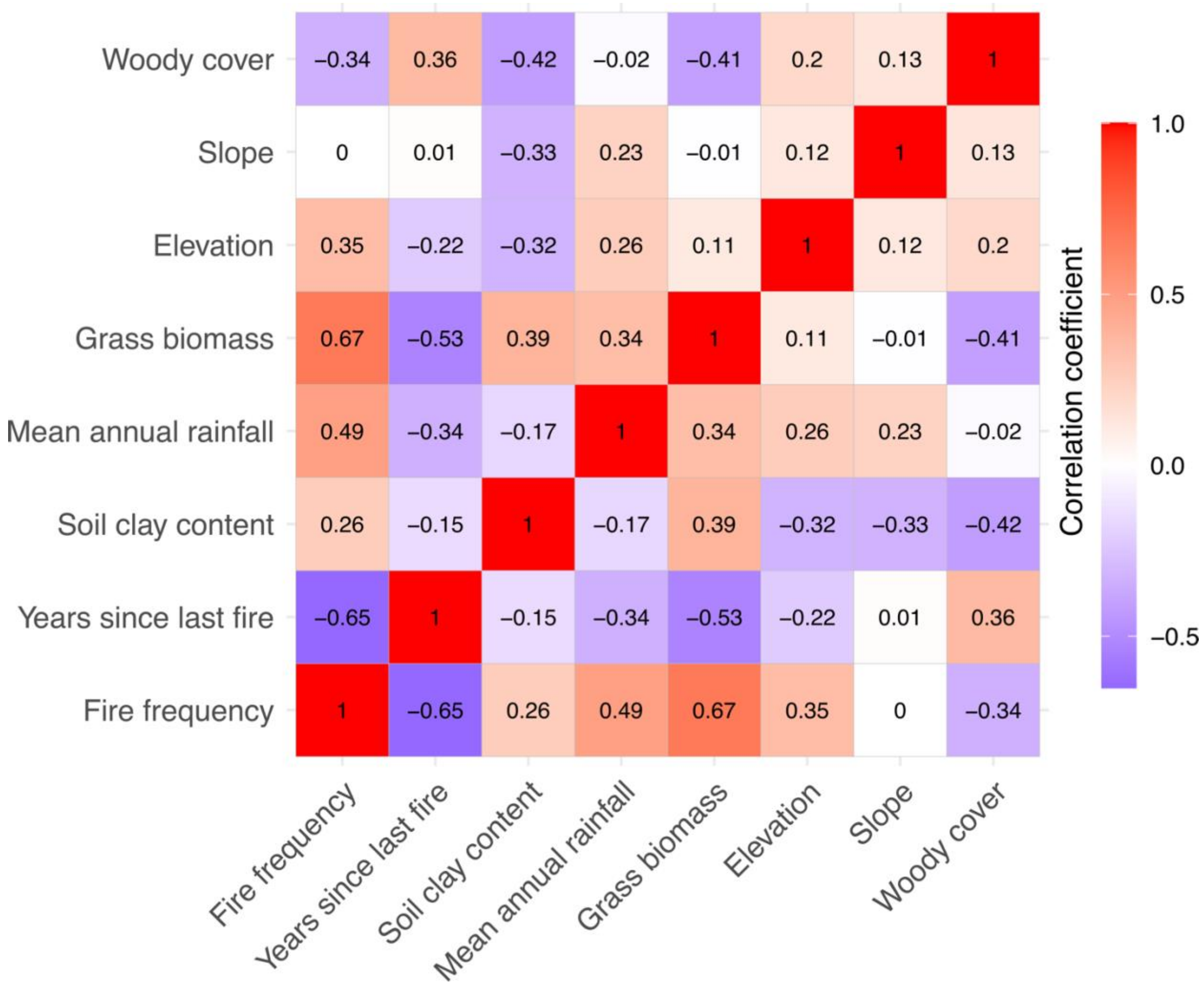

Supplementary Fig. 10: The correlation coefficient matrix among predictors used to model soil pyrogenic carbon accumulation across savannas in Kruger National Park, South Africa

Supplementary Table 1. Generalized linear model specifications and model selection for predicting soil pyrogenic carbon stocks across savannas in Kruger National Park, South Africa.

Intercept	Elevation	Fire Frequency	Grass Biomass	Mean Annual Rainfall	Slope	Soil Clay Content	Woody Cover	Years Since Last Fire	Fire Frequency :Grass Biomass	Fire Frequency : Mean Annual Rainfall	Fire Frequency : Soil Clay Content	Grass Biomass : Mean Annual Rainfall	Grass Biomass: Soil Clay Content	Mean annual Rainfall: Soil Clay Content	df	logLik	AICc	delta	weight
1.179	-0.119	0.125	0.102	-0.284		0.359	-0.054		0.114		-0.090				10	-163.9	349	0	0.023
1.164	-0.134	0.121	0.099	-0.275		0.351	-0.056		0.061	0.055					10	-163.9	349	0.11	0.021
1.183	-0.133	0.142	0.113	-0.287		0.367			0.106		-0.083				9	-165.0	349	0.14	0.021
1.169	-0.147	0.139	0.109	-0.279		0.359			0.058	0.049					9	-165.2	349	0.42	0.018
1.173	-0.134	0.119	0.104	-0.287		0.358	-0.059		0.084	0.039	-0.065				11	-163.0	349	0.5	0.018
1.178	-0.147	0.138	0.115	-0.290		0.367			0.079	0.034	-0.061				10	-164.4	350	1.03	0.014
**1.195**	**-0.170**	**0.143**	**0.107**	**-0.283**		**0.361**				**0.075**					**8**	**-166.7**	**350**	**1.34**	**0.012**
1.176	-0.120	0.129	0.095	-0.283		0.349	-0.055		0.106		-0.111		0.035		11	-163.5	350	1.34	0.012
1.191	-0.159	0.126	0.097	-0.280		0.353	-0.052			0.082					9	-165.7	350	1.37	0.011
1.171	-0.121	0.147	0.102	-0.266		0.355			0.090						8	-166.8	350	1.44	0.011
1.180	-0.118	0.127	0.099	-0.288	0.026	0.367	-0.055		0.112		-0.089				11	-163.6	350	1.52	0.011
1.180	-0.134	0.146	0.106	-0.287		0.357			0.098		-0.103		0.034		10	-164.7	350	1.54	0.011
1.156	-0.143	0.122	0.100	-0.286		0.338	-0.057		0.065	0.053				-0.035	11	-163.6	350	1.56	0.01
1.165	-0.133	0.123	0.095	-0.279	0.025	0.359	-0.057		0.061	0.054					11	-163.6	350	1.66	0.01
1.184	-0.132	0.144	0.110	-0.291	0.025	0.375			0.104		-0.082				10	-164.7	350	1.7	0.01
1.169	-0.136	0.123	0.097	-0.287		0.347	-0.060		0.076	0.040	-0.087		0.037		12	-162.6	351	1.76	0.009
1.165	-0.137	0.124	0.095	-0.280		0.345	-0.059		0.066	0.070		-0.036			11	-163.7	351	1.82	0.009
1.168	-0.108	0.132	0.092	-0.262		0.348	-0.047		0.096						9	-165.9	351	1.88	0.009
1.162	-0.156	0.141	0.111	-0.289		0.347			0.062	0.047				-0.033	10	-164.9	351	1.93	0.009
1.170	-0.146	0.141	0.106	-0.283	0.024	0.367			0.057	0.048					10	-164.9	351	2	0.008

The predictor variables included elevation (m), fire frequency (fires/year), grass biomass (kg/ha), mean annual rainfall (mm), slope (°), soil clay content (%), woody cover (%), years since the last fire (years), and interactions between fire frequency and grass biomass, fire frequency and mean annual rainfall, fire frequency and soil clay content, grass biomass and mean annual rainfall, grass biomass and soil clay content, and mean annual rainfall and soil clay content. The preferred model (the simplest model with $\Delta AIC < 2$) is highlighted in bold and red. The R^2 value for the preferred model in predicting soil pyrogenic carbon stock is 0.60.
